# From suboptimal prey to optimal performance: selective breeding improves *Orius laevigatus* performance on a low-cost mite diet

**DOI:** 10.3389/finsc.2026.1802110

**Published:** 2026-04-27

**Authors:** Amador Rodríguez-Gómez, Virginia Balanza, María del Carmen Reche, Ana Belén Abelaira, Alberto Garre, Pablo Bielza

**Affiliations:** 1 Biocontrol Selection Lab, Departamento de Ingeniería Agronómica, Universidad Politécnica de Cartagena, Cartagena, Spain; 2 Unidad de Microbiología y Seguridad Alimentaria, Departamento de Ingeniería Agronómica, Instituto de Biotecnología Vegetal, Universidad Politécnica de Cartagena, Cartagena, Spain; 3 Instituto de Biotecnología Vegetal, Universidad Politécnica de Cartagena, Cartagena, Spain

**Keywords:** *Acarus siro*, alternative diet, artificial selection, astigmatid mites, biological control, genetic improvement

## Abstract

The mass production of the generalist predator *Orius laevigatus* currently relies on expensive factitious prey, such as *Ephestia kuehniella* eggs. While astigmatid mites offer a low-cost alternative, their use has been historically limited by a significant decline in predator fecundity and fitness. This study evaluates the success of a 15-generation selection program aimed at developed strains adapted to an astigmatid mite diet (*Acarus siro*). We compared two selected lines (MITE1–15 and MITE2-15) against a commercial population. Our results show that the MITE2–15 line overcame the reduced fecundity typically associated with alternative prey, reaching oviposition on *A. siro* comparable to *E. kuehniella*. Furthermore, this genetic gain in reproductive output was achieved without physiological trade-offs, as the selected lines maintained standard morphological size, improved developmental time, and exhibited high survival rates across multiple astigmatid species, including *Suidasia medanensis* and *Carpoglyphus lactis*. A significant population-diet interaction confirms that targeted selection leads to a synergistic adaptation superior to generalist stress-tolerance strategies. These findings represent an advance in the development of improved natural enemies, providing the biocontrol industry with a robust, cost-effective strategy to replace expensive factitious prey without compromising the quality or resilience of the mass-reared predators.

## 1 Introduction

In protected crops, effective augmentative biological control depends on the use of generalist predators to achieve integrated pest management through the consumption of multiple pest species (1, 2). Furthermore, many of these generalist predators are zoophytophagous and their ability to feed on plant resources when prey densities are low allows them to survive and remain in crops (3, 4). Therefore, the constant maintenance of these generalist predators throughout the crop cycle is crucial, as it ensures an immediate biocontrol response in pest infestations (2, 5).

One of the most widely used generalist zoophytophagous insects against small pests in protected crops is *Orius laevigatus* (Fieber) (Hemiptera: Anthocoridae) which is a voracious predator with a broad prey spectrum, effectively attacking spider mites (*Tetranychus* spp.), aphids, whiteflies and particularly different species of thrips, which constitute its primary target. Consequently, it has achieved a prominent biocontrol status in Integrated Pest Management (IPM) programs, especially in greenhouse horticulture, where its commercial releases have drastically reduced the reliance on chemical insecticides (6). This predator is capable of surviving by consuming resources obtained from the plant, such as floral and extra-floral nectar or mesophyll juices. Pollen, however, is the most valuable plant resource, significantly enhancing their fitness and allowing them to persist when natural prey is limited (3, 4). On the other hand, to stimulate the presence of natural enemies when plant resources are not enough in certain crops, supplemental food is often provided artificially in greenhouses, especially in crops with a lack of pollen, such as chrysanthemum or cucumber (7). This strategy often involves expensive alternatives, such as the eggs of *Ephestia kuehniella* (Zeller) (Lepidoptera: Pyralidae), or moderately priced ones like *Artemia franciscana* (Kellogg) (Anostraca: Artemiidae) cysts (8–11), to maintain predator populations preventively (2, 12).

Many authors have studied various alternatives to *E*. *kuehniella* eggs or *Artemia* cysts. Some of these alternative foods, such as artificial diets (based on meat, liver, or egg yolk), have shown a substantial decrease in reproductive fitness when predators are fed on them (13, 14). Similarly, while pollen is widely used for predatory mites, its efficacy as a sole food source for predatory insects is limited due to their low nutritional performance (10, 15). Furthermore, the use of pollen presents its own disadvantages, such as supporting pest species like thrips and susceptibility to fungal attack in warm, humid greenhouse environments (7, 17). On the other hand, a common food source to produce predatory mites is astigmatid mites. These prey mites can be easily and economically mass-produced year-round; however, previous studies employing them as food for predatory insects have generally not been positive, with most studied parameters being lower than when fed optimal diets such as *E. kuehniella* eggs (18–22). This overall nutritional and practical constraint is a major limitation for achieving long-term establishment and is currently recognized as a key challenge in protected crop biological control (23).

One solution to this feeding problem could be the selective breeding of *Orius* predators to improve their adaptation to suboptimal food resources. The ability of omnivorous insects to adapt to different prey suggests a genetic framework, which leads to distinct feeding responses when encountering specific nutritional resources (22, 24–27). This suggests that genetic variation within a species can be manipulated through selection processes to enhance desirable traits (26, 28, 29). Genetic improvement of artificially selected natural enemies has been shown in various studies to be an effective tool to improve relevant phenotypic expression of traits for overcoming biological limitations (23, 30–32). This approach has already proven viable in *O. laevigatus*, with key traits being successfully selected such as insecticide resistance (33–36), increased body size (37, 38), improved response under low temperatures (39) and improved fitness when feeding on pollen (26). Specifically, the study by Mendoza et al. (26) demonstrated that a rigorous selection process could yield strains with a superior capacity to better utilize a suboptimal diet like pollen, which is a cheaper food source than *E. kuehniella* eggs or even *Artemia* cysts. Beyond nutritional adaptation, these selected lines have demonstrated a suite of pleiotropic benefits, including enhanced cold storage tolerance (40) and superior predatory capacity against thrips (38). Furthermore, they exhibit lower intraguild predation (41) and, due to their pollen adaptation, enable significant cost reductions through regulated deficit feeding strategies (42). Despite these multifaceted improvements, the challenge remains to develop a strain that can bridge the fitness gap when fed exclusively on low-cost astigmatid mites.

These studies demonstrated the plasticity of *O*. *laevigatus* to be selected for a suboptimal food, but as mentioned, pollen carries significant disadvantages, which can be detrimental when supplied artificially in crops. On the other hand, recently the same strains selected by Mendoza et al. (26) were evaluated for their performance on different species of astigmatid mites by Rodríguez-Gómez et al. (22), also demonstrating a better response than commercial and wild strains, although without reaching the fitness levels achieved with the optimal *E. kuehniella* eggs diet.

Therefore, taking these previous studies into account, given the economic viability of astigmatid mites and the advantages that can have, such as not causing problems in crops, it is essential to exploit the intraspecific variability of *O. laevigatus* through artificial selection. Building upon recent evidence identifying *Acarus siro* (Linnaeus) (Acari: Acaridae) as the most suitable astigmatid species for this predator (22), developing strains better adapted to this suboptimal food source offers significant potential for both mass rearing and field application. That being said, the specific objectives were: to evaluate the feasibility of enhancing the fitness of *O. laevigatus* when feeding on the astigmatid mite, *A. siro*, by exploiting intraspecific genetic variation through artificial selection; and to assess the biological parameters (survival, development time, longevity, and fecundity) of the selected lines (MITE1–15 and MITE2-15) and a commercial strain when feeding on different astigmatid mite species compared to an optimal diet.

## 2 Materials and methods

### 2.1 Insects rearing

All the populations of *O*. *laevigatus* were reared in the laboratory in 1-L plastic containers with filter paper lids. They were provided ad libitum access to frozen *E. kuehniella* eggs as food, pieces of green bean pods (*Phaseolus vulgaris* L.) as both a moisture source and an oviposition substrate, and buckwheat husk (*Fagopyrum esculentum* Moench) to serve as a shelter and reduce cannibalism. All populations were maintained under controlled environmental conditions of 26 ± 1 °C, 65 ± 5% RH, and a 16:8 h (light:dark) photoperiod.

### 2.2 Establishment of selected lines

We established two new strains, designated MITE1 and MITE2, to select for improved performance on a diet of the astigmatid mite *A. siro*. This species was selected as the target prey because it yielded the highest survival rates and fecundity levels among several astigmatid mites previously evaluated for *O. laevigatus* (22). These new strains were initiated with offspring from a broad genetic base, using individuals from various wild populations, commercial populations, and two previously selected strains (1POL and 2POL) (26).

To create these two distinct genetic lines, eggs were collected from all available source populations and divided into two separate groups to ensure a different genetic foundation for each selection line. Specifically, MITE1 was composed of individuals from the selected lines BIG and 1POL, and the wild populations Pamplona, Málaga, Bres, and Moratalla. MITE2 consisted of the WildMix [a laboratory-stabilized pool of wild populations; Mendoza et al. (26, 38)] and 2POL lines, the commercial population Agrobío, and the wild populations Priañes, Argos, and Faedo [wild populations recently collected; Rodríguez-Gómez et al. (22)]. All founder populations were confirmed to have no prior history of selection or exposure to astigmatid mites. Detailed information regarding the origin and characteristics of these source populations can be found in Mendoza et al. (26, 38) and Rodríguez-Gómez et al. (22).

Once the groups were established, the newly hatched *O. laevigatus* were fed exclusively on *A. siro* throughout their nymphal development. The adult survivors from this initial exposure of each group were then used to initiate the formal selection cycles for each line (MITE1 and MITE2).

### 2.3 Selection protocol

The selection protocol consisted of cycles similar to those described in Mendoza et al. (26). We started by selecting the 200 largest females from the survivors that had been fed on *A. siro* during their development. This selection was based on a visual assessment of overall body volume, a practical approach for handling live individuals. Body size was a key parameter in the selection process, as it serves as a reliable proxy for the nutritional status and the efficiency of diet assimilation in arthropods. Beyond being a critical ecological trait that influences physiology, longevity, and fecundity (37, 43, 44), a larger body size in *O. laevigatus* reflects a superior ability to exploit suboptimal prey. Therefore, selecting for larger individuals ensures that the resulting lines are not only more robust but also more adept at converting the targeted astigmatid mites into biomass. These females were evaluated for their early fecundity (F10), which was measured as the number of eggs laid within the first ten days. Early fecundity was chosen as a selection trait because it has been reported as a good predictor of lifetime fecundity (26, 45, 46). From these 200 females, the top 20% with the highest fecundity were selected, and their offspring completed one selection cycle.

During the selection process, both strains were maintained under the same environmental conditions as the other populations (26 ± 1 °C, 65 ± 5% RH, 16:8 h [light:dark]). After each selection cycle, offspring were reared on *A. siro*. When population size was low, a single multiplication generation was produced on a mixed diet of *A. siro* and *E. kuehniella* eggs to boost numbers, and then eggs from each line was then collected (maximum feasible number), and nymphs were fed exclusively on *A. siro* until adulthood to impose selection on nymphal survival.

After the tenth selection cycle and to guarantee even greater selection pressure, a full-sibling family selection scheme was implemented until the 15th selection cycle (MITE1–15 and MITE2-15). In this phase, the offspring of the 20% most fecund females were reared separately by maternal line and fed exclusively on *A. siro* from eclosion until adulthood. The daughters from each maternal line then underwent the same fecundity-based selection process. Finally, the highest-performing F1 families, based on their reproductive success on the *A. siro* diet, were selected, and their offspring were pooled to establish the next generation.

### 2.4 Populations and diets tested

The biological parameters of the two selected lines (MITE1–15 and MITE2-15) and a commercial population (supplied by Agrobio, Spain) were evaluated. The experiments utilized various species of astigmatid mites as food source: *A. siro*, *Aleuroglyphus ovatus* (Toupeau), *Thyreophagus entomophagus* (Laboulbène), *Tyrolichus casei* (Oudemans), (Acari: Acaridae), *Carpoglyphus lactis* (Linnaeus) (Acari: Carpoglyphidae), and *Suidasia medanensis* (Oudemans) (Acari: Suidasiidae). All mite species were supplied weekly by Agrobio (Spain) and were maintained separately on wheat bran used as a feeding substrate for all life stages and for all mite cultures, following the same maintenance conditions as in Rodríguez-Gómez et al. (22). Each species was kept in individual bottles at 15 ± 1 °C and 65 ± 5% RH. A diet of *E. kuehniella* eggs was used as an optimal control diet.

### 2.5 Evaluation of development time, survival, and body size

To obtain fresh eggs for each population, small segments of green bean pods were placed in adult-rearing containers, replaced daily for one week, and then stored at a low temperature of 6 °C to prevent embryonic development. Preliminary trials conducted under our laboratory established that the viability and subsequent growth of the embryos were not compromised by storing oviposition pods at 6 °C for up to 10 days. Consequently, for the present experiments, eggs were kept at this temperature for a maximum of 7 days. The high survival rates obtained (see Results) further validate that this storage protocol did not induce sublethal effects on embryonic development. Egg quantification was performed under a stereoscopic microscope, and the exact number of eggs (ranging between 80–100) was recorded for each 200 ml cardboard cup replicate to allow for precise survival calculations. Each cup contained a small bean pod, buckwheat husk, and the designated diet. We prepared five replicates for each population and diet, using *E. kuehniella* as a reference control. To ensure food quality and humidity, bean pods and prey were replaced three times per week. In all cases, prey was provided *ad libitum* to ensure that nutritional availability was not a limiting factor during nymphal development.

Throughout the nymphal development, the final survival rate was calculated as the percentage of initial eggs that successfully developed to adults. Developmental time was determined by daily monitoring of newly emerged adults. Starting from day 8, adults less than 24 hours old were removed daily until no nymphs remained. The mean interval between the egg stage and adult emergence was recorded per replicate. In this sense, the cup served as the independent experimental unit. Additionally, emerging adults were sexed, and their maximum pronotum width was measured (after freezing) with an optical micrometer at 50× magnification. The experiment was conducted under controlled conditions of 26 ± 1 °C, 65 ± 5% RH, and a 16:8 h (light:dark) photoperiod.

### 2.6 Fecundity and longevity assessment

Based on the superior performance observed in the previous trial, this experiment focused exclusively on the MITE2–15 line and the commercial population (Agrobio). As diets, three prey were provided: *A. siro*, the species for which the line had been selected; *C. lactis*; and *E. kuehniella*, which served as in the previous experiment as the optimal diet and positive control. The species *C. lactis* was specifically included to determine if the reproductive advantages were diet-specific or stemmed from a genetic improvement of the population, given that this mite showed lower performance in the previous survival trial and in the study by Rodríguez-Gómez et al. (22). To assess reproductive traits, fifth-instar nymphs of both the MITE2–15 and Agrobio populations were maintained on an *E. kuehniella* diet until reaching maturity. Four days post-emergence, 30 mated females per group were individually isolated in 30 mL cups, with each female serving as a single replicate (n = 30). These units contained the respective test diets and a 3 cm bean pod section as an oviposition substrate.

Monitoring was conducted twice weekly using a stereomicroscope to count eggs and refresh both the food and the bean pods. During these sessions, females were provided with their respective diets *ad libitum*. These procedures continued throughout the females entire lifespan to determine cumulative fecundity and longevity. To account for potential nutritional carry-over from the juvenile stage, the first oviposition data (days 3–4) were omitted from the analysis. Fecundity means were calculated across all females, including those that laid zero eggs. All trials were performed under the same controlled conditions described for the survival experiments.

### 2.7 Statistical analysis

To evaluate the impact of population and prey, a two-way factorial design was implemented. Developmental time, body size and longevity were analyzed using a two-way factorial Analysis of Variance (ANOVA) after confirming that residuals followed a normal distribution (Shapiro-Wilk test) and variances were homogeneous (Levene’s test). No data transformations were required, as the assumptions of normality and homoscedasticity were met for these variables analyzed. In contrast, fecundity and survival were addressed through GLMs to better handle the nature of the data. Specifically, a Negative Binomial GLM was chosen for egg counts to manage overdispersion, while survival proportions were fitted to a Binomial GLM.

Model optimization for each GLM was guided by the Akaike Information Criterion (AIC), comparing additive, interaction and main effects models. The statistical importance of factors was determined via Likelihood Ratio (LR) χ^2^ tests. When significant differences were detected, means were separated using Tukey’s HSD *post-hoc* tests (with Šídák adjustment for interactions). Results throughout the study are expressed as mean ± SE, with a threshold for significance of *p* < 0.05. All statistical analyses were conducted using R software (version 4.5.2 (47);). For the Negative Binomial GLM the ‘MASS’ package was used (48).

## 3 Results

### 3.1 Immature survival, developmental time and body size

The survival analysis based on the AIC favored the interaction model (AIC = 1567.2) over the additive model (AIC = 1735.1), indicating that the effect of the prey was not uniform across populations. There were highly significant effects for population (χ^2^ = 1064.3, df = 2, *p* < 0.001), prey type (χ^2^ = 1937.71, df = 6, *p* < 0.001), and their interaction (χ^2^ = 192.01, df = 12, *p* < 0.001). Between populations, significant differences were found; specifically, both selected strains (MITE1–15 and MITE2-15) exhibited significantly greater survival than the commercial (Agrobio) population, with MITE2–15 showing in general the highest survival across all tested diets. Regarding the prey provided, while overall survival was highest for all populations when fed *E*. *kuehniella* eggs, the selected lines significantly outperformed the commercial strain on all tested suboptimal mite species. Notably, the survival rates of the selected populations (MITE1–15 and MITE2-15) when feeding on *T. entomophagus* or *A. siro* were statistically comparable to those achieved on the optimal *E. kuehniella* eggs diet (**Table 1**).

**Table 1:** Mean ( ± SE) survival from egg to adult (%), developmental times (days) and body sizes (pronotum width of females and males in mm) of three populations of *O. laevigatus*, Agrobio, MITE1–15 and MITE2–15 fed with different prey (*E. kuehniella* eggs and different species of astigmatid mites).

Prey	Population	% Survival			Development time (days)			Pronotum width female (mm)			Pronotum width male (mm)		
*E. kuehniella*	Agrobio	90.9	± 3.6	ab	12.4	± 0.06	Da	0.80	± 0.0026	Aa	0.75	± 0.0034	Aa
	MITE1-15	95.8	± 1.3	a	12.3	± 0.13	Db	0.79	± 0.0027	Aa	0.75	± 0.0025	Aa
	MITE2-15	91.5	± 4.7	ab	11.7	± 0.11	Dc	0.79	± 0.0029	Aa	0.74	± 0.0020	Aa
*A. siro*	Agrobio	48.4	± 10.0	gh	12.3	± 0.10	Da	0.77	± 0.0028	Ba	0.74	± 0.0035	Ba
	MITE1-15	75.9	± 2.6	cd	12.3	± 0.03	Db	0.76	± 0.0027	Ba	0.73	± 0.0022	Ba
	MITE2-15	86.6	± 11.3	b	12.0	± 0.26	Dc	0.76	± 0.0019	Ba	0.73	± 0.0019	Ba
*C. lactis*	Agrobio	28.3	± 3.1	jk	16.3	± 0.19	Aa	0.70	± 0.0037	Ea	0.65	± 0.0044	Fa
	MITE1-15	65.4	± 7.9	ef	17.4	± 0.31	Ab	0.71	± 0.0046	Ea	0.66	± 0.0046	Fa
	MITE2-15	76.5	± 9.6	cd	15.7	± 0.28	Ac	0.71	± 0.0039	Ea	0.66	± 0.0048	Fa
*T. entomophagus*	Agrobio	49.1	± 6.5	gh	12.8	± 0.19	Da	0.73	± 0.0032	Da	0.70	± 0.0034	Ea
	MITE1-15	87.1	± 3.8	b	12.0	± 0.21	Db	0.73	± 0.0021	Da	0.70	± 0.0023	Ea
	MITE2-15	95.8	± 1.0	a	12.2	± 0.19	Dc	0.73	± 0.0034	Da	0.70	± 0.0032	Ea
*A. ovatus*	Agrobio	49.6	± 4.1	gh	12.9	± 0.08	Da	0.75	± 0.0026	Ca	0.72	± 0.0032	Da
	MITE1-15	72.3	± 5.3	de	12.2	± 0.12	Db	0.74	± 0.0021	Ca	0.71	± 0.0026	Da
	MITE2-15	85.5	± 6.5	bc	12.3	± 0.19	Dc	0.75	± 0.0022	Ca	0.71	± 0.0024	Da
*T. casei*	Agrobio	22.0	± 9.1	k	14.6	± 0.16	Ca	0.76	± 0.0036	Ba	0.73	± 0.0046	Ca
	MITE1-15	34.5	± 15.0	ij	14.0	± 0.39	Cb	0.76	± 0.0067	Ba	0.72	± 0.0073	Ca
	MITE2-15	37.2	± 3.4	ij	13.0	± 0.05	Cc	0.76	± 0.0050	Ba	0.72	± 0.0038	Ca
*S. medanensis*	Agrobio	7.7	± 1.2	l	15.4	± 0.17	Ba	0.69	± 0.0052	Fa	0.63	± 0.0084	Ga
	MITE1-15	40.9	± 7.4	hi	15.2	± 0.26	Bb	0.67	± 0.0036	Fa	0.63	± 0.0040	Ga
	MITE2-15	58.2	± 12.7	fg	15.2	± 0.24	Bc	0.67	± 0.0043	Fa	0.63	± 0.0043	Ga

Comparisons of means within a column are denoted by differing letters: uppercase letters signify a significant main effect of the prey as a food source (same letter for all populations within each prey) and lowercase letters indicate a significant main effect of the *O. laevigatus* population (same letter for all prey within each population) (*p* > 0.05; Tukey *post hoc* test). When the interaction between prey and population is significant (survival), means are compared pairwise within column (*p* < 0.05; Šídák’s *post hoc* test).

As also shown in **Table 1**, the development time from birth to adulthood showed significant differences concerning both the prey factor (F = 218.15, df = 6/80, *p* < 0.001) and the population factor (F = 23.58, df = 2/80, *p* < 0.001), though no significant differences were found for the interaction between these factors (F = 1.80, df = 12/80, *p* > 0.05). Specifically, among the *O. laevigatus* populations tested, both selected lines (MITE1–15 and MITE2-15) achieved significantly shorter development times than the commercial Agrobio population; furthermore, between the two, MITE2–15 outperformed MITE1-15, exhibiting the overall fastest development. The development period was significantly shorter when *O. laevigatus* were fed *E. kuehniella* eggs (12 days), *T. entomophagus* (12 days), or *A. siro* (12 days), in comparison to the other prey items used as diet; specifically, *C. lactis* resulted in the longest development time among all diets tested (16 days).

The ANOVA analysis of the pronotal sizes for females and males (**Table 1**) revealed that prey was the sole statistically significant factor influencing size, demonstrating a strong effect on both females (F = 364.52, df = 6/1871, *p* < 0.001) and males (F = 334.63, df = 6/1736, *p* < 0.001). Conversely, no significant differences were found with respect to the population factor (Females: F = 2.07, df = 2/1871, *p* > 0.05; Males: F = 0.44, df = 2/1736, *p* > 0.05), nor was the interaction between the two factors significant (Females: F = 0.98, df = 12/1871, *p* > 0.05; Males: F = 1.23, df = 12/1736, *p* > 0.05). Across both sexes, the largest average size was consistently achieved when individuals were fed *E. kuehniella* eggs (Females: 0.79 mm; Males: 0.75 mm), followed by those individuals fed with *A. siro* (Females: 0.76 mm; Males: 0.73 mm), and the smallest average size occurred when fed *S. medanensis* (Females: 0.67 mm; Males: 0.63 mm).

### 3.2 Female fecundity and longevity

In fecundity analysis the model selection based on AIC favored the interaction term (AIC = 1592.2), as it provided a better fit than the additive model (AIC = 6283.6). The analysis confirmed that fecundity was significantly influenced by both the main effects of population and prey type (χ^2^ = 20.75, df = 1, *p* < 0.001 and χ^2^ = 27.00, df = 2, *p* < 0.001, respectively), as well as their interaction (χ^2^ = 13.39, df = 2, *p* = 0.0012). The selected MITE2–15 population exhibited significantly higher fecundity compared to the commercial population across all diets. Specifically, MITE2–15 females provided with *E. kuehniella* eggs demonstrated the highest fecundity (60 eggs). Notably, when fed with the astigmatid mite *A. siro*, MITE2–15 females achieved a fecundity of 58 eggs, a value nearly identical to that recorded on the optimal *E. kuehniella* diet. Even when feeding on a species of astigmatid mite such as *C. lactis*, which had previously had relatively low fecundities in Rodríguez-Gómez et al. (22), MITE2–15 showed considerably high fecundity (29 eggs). This superior performance was even more pronounced when compared to the commercial population, which registered 24 and 5 eggs when fed *A. siro* and *C. lactis*, respectively (**Table 2**).

**Table 2:** Results of experiments (fecundity and longevity) with two populations of *O. laevigatus*, Agrobio and MITE2–15 fed with different prey (*E. kuehniella* eggs and different species of astigmatid mites).

Prey	Population	Fecundity (eggs/female)			Longevity (days)		
*E. kuehniella*	Agrobio	55.3	± 10.7	ab	17.7	± 2.1	Aa
	MITE2-15	60.2	± 8.3	a	15.2	± 1.5	Aa
*A. siro*	Agrobio	24.2	± 3.3	b	12.8	± 0.8	Aa
	MITE2-15	58.0	± 6.2	ab	16.7	± 1.3	Aa
*C. lactis*	Agrobio	5.0	± 1.1	c	8.9	± 0.8	Ba
	MITE2-15	29.7	± 3.0	ab	9.3	± 0.9	Ba

Comparisons of means within a column are denoted by differing letters: uppercase letters signify a significant main effect of the prey as a food source (same letter for all populations within each prey) and lowercase letters indicate a significant main effect of the *O. laevigatus* population (same letter for all prey within each population) (*p* > 0.05; Tukey *post hoc* test). When the interaction between prey and population is significant (fecundity), means are compared pairwise within column (*p* < 0.05; Šídák’s *post hoc* test).

Pertaining to female longevity in the commercial Agrobio population and MITE2-15, results are presented also in **Table 2**. The prey factor showed statistically significant effects on longevity (F = 16.97, df = 2/174, *p* < 0.001). Females fed with *E. kuehniella* eggs and *A. siro* resulted in the highest longevity, significantly different from the prey *C. lactis*. In contrast, the population factor (F = 0.29, df = 1/174, *p* > 0.05) and the interaction between population and prey were not statistically significant (F = 3.02, df = 2/174, *p* > 0.05).

## 4 Discussion

The development of genetically improved strains of natural enemies is essential for advancing augmentative biological control, particularly in addressing the challenge of prey-density dependence in protected crops (23, 26). In this study, a rigorous selection process led to the creation of strains that represent a significant breakthrough in nutritional adaptation to a suboptimal food source, such as astigmatid mites. To date, the use of these mites has yielded controversial performance in *O. laevigatus* (22). However, our results demonstrate that the fitness gap between high-quality factitious prey (*E. kuehniella* eggs) and low-cost alternative diets can be virtually eliminated through targeted genetic improvement.

A recurrent theme in recent literature regarding *O. laevigatus* is the search for alternative diets that can substitute the expensive factitious diet of *E. kuehniella* eggs (13–16). However, Mendoza et al. (26) proposed a different solution: instead of the constant search for new alternative foods, they advocated for the genetic improvement of strains selected for suboptimal diets. Despite this, a dichotomy between juvenile survival and adult reproduction often persists when using alternative prey. Rodríguez-Gómez et al. (22) observed that when *O. laevigatus* strains previously selected for pollen by Mendoza et al. (26) (1POL and 2POL) were fed certain astigmatid mites like *A. siro*, they maintained nymphal survival rates comparable to the benchmark diet; however, fecundity dropped from 70 to an average of 40 eggs per female. Mendoza et al. (26) emphasized that omnivorous predators often experience a significant decline in fitness when feeding on non-prey or suboptimal foods, noting a 60–67% reduction in fecundity when switching from *E. kuehniella* eggs to pollen. While their selected lines (1POL-13 and 2POL-11) successfully doubled fecundity on pollen compared to reference populations, they still did not reach the fecundity levels achieved with optimal prey.

Our findings with the strain specifically selected for astigmatid mites (MITE2-15) challenge this consensus. By reaching a fecundity of 58 eggs on *A. siro*—statistically indistinguishable from the 60 eggs on *E. kuehniella*, we demonstrate that the decline in fecundity observed by Mendoza et al. (26) and Rodríguez-Gómez et al. (22) is not an inherent biological limitation, but rather a reflection of untapped genetic potential. The fact that our MITE2–15 line reached fecundity levels similar to those of the optimal diet confirms, through our significant interaction model, a synergistic adaptation between the MITE2–15 genotype and the *A. siro* diet, representing a step beyond simple additive improvement.

This achievement is even more significant when considering that wild or commercial populations of *O. laevigatus* have shown very low survival rates when feeding on various astigmatid mite species compared to *E. kuehniella* (22). In contrast, our specifically selected lines (MITE1–15 and MITE2-15) achieve survival percentages previously seen only in other species of the genus. For example, Husseini et al. (18) reported that *O. majusculus* can reach 90% survival on *Tyrophagus putrescentiae*, and Bernardo et al. (20) observed 87% survival for *O. insidiosus*. Furthermore, Bonte et al. (21) highlighted drastic interspecific variation: while *O. thripoborus* survived well on *C. lactis* (54%), *O. naivashae* exhibited survival rates near zero. Our MITE lines bridge this interspecific gap, providing a robust performance that extends beyond a single prey species.

Moreover, our study reveals that selection focused on a single astigmatid species (*A. siro*) can confer a broader multispecies resilience. Rodríguez-Gómez et al. (22) reported that survival varies drastically depending on the mite species, with extremely low results on *S. medanensis* or *T. casei*. Conversely, the MITE1–15 and MITE2–15 lines showed significantly higher survival across different astigmatid species, such as *S. medanensis* and *C. lactis*. This suggests that the selection process did not produce a narrow specialist, but rather a more efficient generalist capable of better exploiting the available nutrients in different species of prey.

Physiologically, the selected lines appear to have optimized their metabolic conversion of mite-derived nutrients. This is evidenced by their faster development (12 days) compared to the commercial strain and greater adult fecundity when facing challenging mites like *C. lactis*. The absence of costs in longevity or body size, as both males and females maintain sizes equal to the standard population, further supports the idea of a shift in the fitness frontier, as previously hypothesized for other selected *Orius* strains (26, 38). While body size is significantly reduced when fed on astigmatid diets compared to *E. kuehniella* eggs, this does not impair traits such as development time or survival in our selected lines. This lack of adaptation costs contradicts traditional allocation theory (49), which suggests that increasing reproductive effort on deficient diets should shorten lifespan. Instead, our findings align with the idea that genetic improvement can completely modify the adaptive frontier, allowing for a strain that matures faster, lives longer, and reproduces more efficiently on low-cost resources.

Future research should focus on validating these findings under industrial mass-rearing and field conditions, as laboratory conditions may not fully reflect the dynamic fluctuations in temperature and humidity in other fields. Further research should also focus on long-term studies assessing multi-generational responses entirely on mite diets (9). In addition, it will be essential to evaluate whether prolonged selection on *A. siro* affects key ecological traits such as predatory efficiency, prey preference, and functional response toward natural prey species. Previous work with O. laevigatus selected on alternative diets (e.g., pollen-adapted lines) has shown that such selection does not necessarily impair, and may even enhance, predatory performance on target pests (38), suggesting a high degree of behavioral plasticity in this species. Nevertheless, targeted assays on natural prey will be required to confirm that the selected MITE lines maintain full field efficacy after long-term rearing on astigmatid mites. Nevertheless, the evidence suggests that the MITE selected lines represent a more sustainable and economically biological control strategy. Validating the suitability of *A. siro* as a total replacement for *E. kuehniella* in semi-field conditions will be the next crucial step to ensure that this nutritional fitness translates into superior pest control and enhanced economic viability for biofactories.

In conclusion, this study demonstrates that targeted genetic selection can effectively overcome the nutritional barriers that traditionally limit the use of low-cost alternative prey in *O. laevigatus*. Our MITE2–15 line not only outperformed commercial strains but also achieved reproductive performance in *A. siro* statistically equivalent to that of the factitious food *E. kuehniella* eggs. By overcoming limitations in fecundity without sacrificing physiology trade-offs, these selected lines provide a cost-effective, high-performance solution for mass rearing or integrated biological control programs. These lines offer a way to reduce production costs while ensuring the release of resilient predators, marking a step forward in the development of more optimized natural enemies for sustainable integrated pest management.

## Data Availability

The raw data supporting the conclusions of this article will bemade available by the authors, without undue reservation.
